# Identification of Virulent Capnocytophaga canimorsus Isolates by Capsular Typing

**DOI:** 10.1128/JCM.00249-17

**Published:** 2017-05-23

**Authors:** Estelle Hess, Francesco Renzi, Dunia Koudad, Mélanie Dol, Guy R. Cornelis

**Affiliations:** Unité de Recherche en Biologie des Microorganismes, Université de Namur, Namur, Belgium; Mayo Clinic

**Keywords:** capsular polysaccharide, ELISA, PCR, septicemia, serotyping scheme, Western blot

## Abstract

Capnocytophaga canimorsus is a dog oral commensal that causes rare but severe infections in humans. C. canimorsus was recently shown to be endowed with a capsular polysaccharide implicated in resistance to the innate immune system of the host. Here, we developed the first C. canimorsus capsular serotyping scheme. We describe nine different serovars (A to I), and this serotyping scheme allowed typing of 25/25 isolates from human infections but only 18/52 isolates from dog mouths, indicating that the repertoire of capsules in the species is vast. However, while only three serovars (A, B, and C) covered 88% of the human isolates tested (22/25), they covered only 7.7% of the dog isolates (4/52). Serovars A, B, and C were found 22.9-, 14.6-, and 4.2-fold more often, respectively, among human isolates than among dog isolates, with no geographical bias, implying that isolates endowed with these three capsular types are more virulent for humans than other isolates. Capsular serotyping would thus allow identification of virulent isolates in dogs, which could contribute to the prevention of these infections. To this end, we developed a PCR typing method based on the amplification of specific capsular genes.

## INTRODUCTION

Capnocytophaga canimorsus is an agent of septicemia that often evolves to septic shock in spite of adequate treatment (1). Since its discovery in 1961 (2), more than 480 cases of infections were reported in the literature (for a recent review, see reference 3). With a mortality rate of 30% and significant morbidity, the prognosis of C. canimorsus sepsis is poor (4, 5). Although less frequently reported, meningitis and endocarditis are also associated with C. canimorsus infections (3). The genus Capnocytophaga, which belongs to the family Flavobacteriaceae in the phylum Bacteroidetes, comprises capnophilic species found in the oral cavities of humans and domestic animals. The mouths of dogs and cats host C. canimorsus (formerly dysgonic fermenter-2 [DF-2] [6]), Capnocytophaga cynodegmi (1, 6), and the newly described Capnocytophaga canis (7) and “Capnocytophaga stomatis” (8), but only C. canimorsus is associated with severe human infections (1, 7, 8). According to studies carried out in different countries, the prevalence of C. canimorsus ranges from 19 to 74% in dogs and 21 to 57% in cats (9–13). However, these figures may include C. canis and C. stomatis, which were recently separated from the C. canimorsus species. Transmission to humans mostly occurs through dog (97%) or cat (3%) bites, scratches, licks, or simple contact (3, 14). The prevalences of C. canimorsus infections were estimated at 0.5 and 0.63 cases per million inhabitants per year in Denmark (5) and in the Netherlands (15), respectively, but a recent study in the Helsinki area (Finland) estimated the prevalence to be as high as 4.1 cases per million inhabitants per year (16). C. canimorsus infections could thus be underdiagnosed due to the fastidious and slow growth of C. canimorsus in culture (1, 17). In addition, the initial clinical manifestations of C. canimorsus infections are not specific, and their onset can be as late as 8 days after contact with a dog (3, 5). The median age of patients is between 52 and 59 years, and a male-to-female ratio of 3/2 is generally observed (3, 5, 16). Splenectomy and alcohol abuse are common predisposing factors, but up to 40% of patients presented no obvious risk factor (18), implying that C. canimorsus cannot solely be considered an opportunistic pathogen.

C. canimorsus strain 5 (Cc5, BCCM/LMG 28512), a strain isolated from a fatal septicemia case (19), has a lipooligosaccharide (LOS) and a capsular polysaccharide (CPS) which are genetically and biochemically related (20). The CPS plays a key role in innate immunity evasion by conferring Cc5 its resistance to phagocytosis by macrophages, to polymyxin B, and to 10% human serum (20). In addition to being recognized virulence factors for both Gram-negative and Gram-positive bacteria (for a review, see reference 21), CPS are also useful to serotype bacteria and to identify virulent isolates (22, 23). Here, we show that 25 isolates of C. canimorsus out of 25 from a collection of isolates from human infections are endowed with a CPS and that those polysaccharide structures present limited variability, with 3 dominant capsular serovars. In addition, a clear enrichment of these dominant capsular serovars was found in human isolates (22/25) compared to isolates from dog mouths (4/52). Finally, we show that PCR typing can be used to detect these serovars that are more virulent in humans. This study paves the way toward prevention of these dramatic infections.

## RESULTS

### Capsular serotyping identifies 5 serovars in a collection of C. canimorsus isolates from human infections.

**FIG 1:**
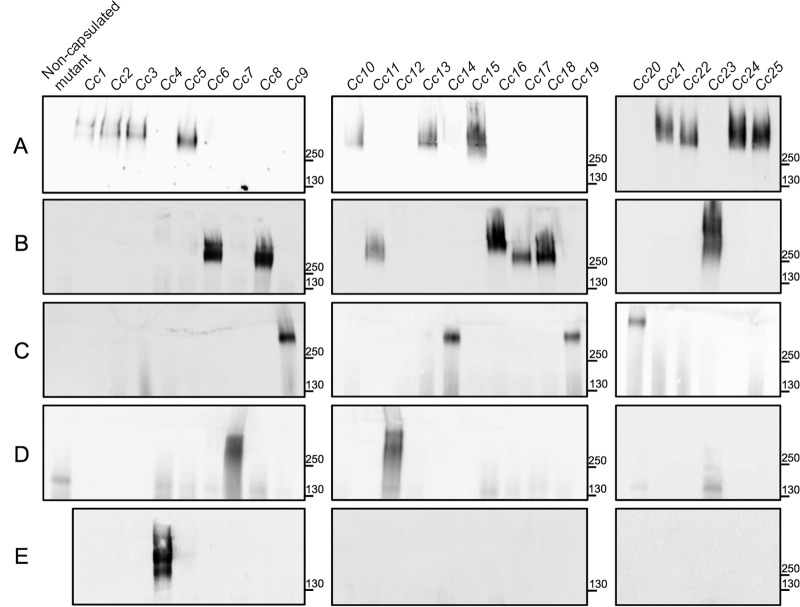
Capsular serotyping of C. canimorsus isolates from human infections. Western blot analysis of proteinase K-treated lysates of C. canimorsus human isolates using the following sera: Y1C12-adsorbed anti-Cc5 (A), anti-Cc6 (B), anti-Cc9 (C), anti-Cc12 (D), and anti-Cc4 (E). Noncapsulated Cc5 Y1C12, Cc6 Δ*wbuB*, Cc9 Δ*wbuB*, and Cc12 Δ*wbtA* mutants were used as controls in panels A, B, C, and D, respectively. Numbers correspond to molecular weight markers in thousands.

The prevalence of the capsular serovar of strain Cc5 was tested in a collection of 25 C. canimorsus isolates from human infections (Table S1). Whole bacteria were digested with proteinase K, and bacterial polysaccharides were analyzed by Western blotting using an antiserum directed against Cc5 bacteria and adsorbed with the noncapsulated Cc5 transposon mutant Y1C12 (20, 24). The serum recognized a high-molecular-weight (HMW) band (>250,000) in the extracts from Cc5 and from 10 other isolates, namely, Cc1 (BCCM/LMG 11511, CCUG 17234, strain P810, strain SSI P810), Cc2, Cc3, Cc10 (BCCM/LMG 11541, CCUG 24741, ATCC 35978, CDC C8936), Cc13, Cc15, Cc21 (CCUG 60839), Cc22 (CCUG 20318), Cc24 (CCUG 67384), and Cc25 (CCUG 66222) (Fig. 1A). Since this band was identified as the CPS of Cc5 (20), we concluded that the capsular serovar of Cc5 was shared with these 10 isolates, representing 44% of our collection of human isolates. We named this capsular serovar A.

To determine the capsular serovar(s) of the 14 non-A human isolates, 9 new antisera were raised and tested by Western blot on polysaccharide extracts from these 14 isolates. The antisera raised against Cc6, Cc9 (BCCM/LMG 11510, CCUG 12569, CDC A3626), Cc12 (type strain, ATCC 35979, CDC7120, CCUG 53895), and Cc4 allowed the detection of a HMW polysaccharide, most likely corresponding to a CPS (Fig. 1) in all 14 isolates. The anti-Cc6 serum recognized a HMW polysaccharide structure in Cc6 but also in Cc8, Cc11, Cc16, Cc17, Cc18, and Cc23 (CCUG 48899) (Fig. 1B). This serovar, named B, thus had a prevalence of 28% in our collection of human isolates, with 7 isolates positive out of 25. The anti-Cc9 serum recognized a HMW polysaccharide structure in Cc9, Cc14, Cc19, and Cc20 (CCUG 55909) (Fig. 1C). This serovar, named C, thus had a prevalence of 16%. The anti-Cc12 serum recognized a HMW polysaccharide structure in Cc12 and Cc7 (Fig. 1D). The prevalence of this serovar, named D, was 8%, thus showing that it is more limited than serovars A, B, and C. Finally, the anti-Cc4 serum recognized a HMW polysaccharide band only in Cc4. This serovar thus had a prevalence of only 4% and was named E (Fig. 1E).

In order to confirm that the HMW bands recognized are CPS, we next attempted to generate noncapsulated deletion mutants of Cc6, Cc9, Cc12, and Cc4 (Table S2). Since the capsule of Cc5 is made of the same sugars as the LOS O chain, we decided to generate rough noncapsulated mutants. To this aim, we sequenced the genomes of isolates Cc6, Cc9, and Cc4 and used the previously published genome sequence of Cc5 (GenBank accession no. CP002113) (25) and draft genome sequence of Cc12 (GenBank accession no. CDOE00000000.1) (26). Homologs of the Cc5 *wbuB* gene (*Ccan_23370*), which is the gene mutated in the LOS/CPS mutant Y1C12 and encodes an *N*-acetyl-fucosamine (FucNAc) transferase (20), were found in the genomes of Cc6 (*CC6_1430029*) and Cc9 (*CCAN9_740038*) but not in those of Cc12 and Cc4. In the genomes of Cc12 and Cc4, we identified homologs of Cc5 *wbtA* (*Ccan_23400*) that is mutated in the LOS/CPS mutant Y1D1 (20) of Cc5 and encodes an UDP-*N*-acetylglucosamine 4,6-dehydratase (*CCAN12_760057*, and *CC4_530070*, respectively) (20). The *wbuB* genes were thus mutated in Cc6 and Cc9, while gene *wbtA* was mutated in Cc12. The polysaccharide extracts from the mutants of Cc6, Cc9, and Cc12, analyzed by Western blotting with the anti-Cc6, anti-Cc9, and anti-Cc12 sera, did not contain the HMW band, indicating that it was indeed a CPS (Fig. 1B to D). Gene *wbtA* from Cc4 could not be mutated, despite several attempts; hence, we could not formally prove that the HMW polysaccharide is a CPS related to the LOS. Nevertheless, the presence of a *wza* homolog encoding the capsular transporter across the outer membrane suggests that Cc4 is indeed endowed with a capsule.

We thus conclude that the 25 C. canimorsus human isolates of our collection are all endowed with a CPS and that the antigenic repertoire of these CPS is limited, given that 88% of the isolates (22/25) belong to serovars A, B, and C. Interestingly, the distribution of serovars A, B, and C is not affected by a geographical bias, since each serovar was found in isolates from at least three different countries (Table S5).

### Prevalence rates of capsular serovars A, B, and C among C. canimorsus isolated from dog mouths.

**FIG 2:**
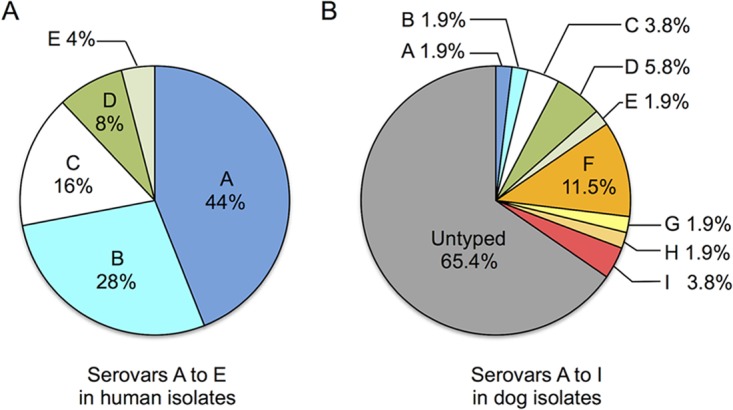
Prevalences of capsular serovars A to I in C. canimorsus isolates from human infections (A) and dog mouths (B).

We next assessed the prevalences of the capsular serovars A to E among a collection of 52 isolates of C. canimorsus from dog mouths (Table S1) (7). To this aim, we set up an enzyme-linked immunosorbent assay (ELISA) screening using entire heat-killed bacteria. Since we needed sera that specifically recognized the CPS, for serovar A, we used the Y1C12-adsorbed anti-Cc5 serum; for serovars B, C, and D, we adsorbed the crude anti-Cc6, anti-Cc9, and anti-Cc12 sera with Cc6 *wbuB*, Cc9 *wbuB*, and Cc12 *wbtA* mutant bacteria, respectively. Due to the lack of a noncapsulated Cc4 mutant strain, we adsorbed the anti-Cc4 serum (serovar E) with the 24 other human isolates belonging to different capsular serovars (Fig. 1E). The efficacies of the different adsorptions were validated by immunofluorescence staining and microscopy analysis (Fig. S1). The five adsorbed sera were then used to test our collection of dog isolates by ELISA. The reactivity of each isolate was calculated with respect to that of the type strain of each serovar (Cc5 for A, Cc6 for B, Cc9 for C, Cc12 for D, and Cc4 for E). The noncapsulated mutant strains were used as negative controls. The results of the screening are summarized in Table 1. Only two isolates, CcD68 and CcD105, were positive for serovar A, with reactivities of 43% ± 7% and 107% ± 28%, respectively. The HMW polysaccharide structures of these isolates were analyzed by Western blotting, and only the strongly reacting CcD105 displayed a serovar A capsule (Fig. S2A). For serovar B, only isolate CcD68 was found to be positive (110% ± 11%) by ELISA and by Western blotting (Fig. S2B). For serovar C, isolates CcD43 and CcD130 were positive by ELISA (86% ± 5% and 108% ± 26% reactivities, respectively) and Western blotting (Fig. S2C). For serovar D, three isolates were strongly recognized by ELISA and confirmed by Western blotting: CcD16 (86% ± 14%), CcD89 (95% ± 9%), and CcD117 (99% ± 12%) (Fig. S2D). Finally, for serovar E, isolate CcD96 displayed a high reactivity of 118% ± 37%, and isolates CcD20 and CcD106 displayed intermediate reactivities of 57% ± 24% and 59% ± 24%, respectively, while some other isolates presented limited reactivity. All the isolates with a value equal or higher than 30% were checked by Western blotting, and only one isolate, CcD96, was confirmed to belong to serovar E (Fig. S2E). The results from the ELISA and the Western blot analyses are summarized in Fig. 2. While all the human isolates belonged to serovars A, B, C, D, or E, 84.6% of the dog isolates were left nontypeable. In conclusion, the prevalence of serovar A was 22.9-fold higher in human isolates than in dog isolates (Fisher's exact test, *P* = 6.45 × 10^−6^), while the prevalence of serovar B was 14.6-fold higher (Fisher's exact test, *P* = 0.00123). A 4.2-fold increase was found for the serovar C, but it was not statistically significant (Fisher's exact test, *P* = 0.0831). Finally, there was no significant difference in the prevalences of serovars D and E (*P* = 0.657 and 0.547, respectively, Fisher's exact test).

**TABLE 1:** Capsular serotyping of C. canimorsus dog isolates by ELISA

Strain/isolate	Capsular serovar^*a*^								
	A	B	C	D	E	F	G	H	I
**Cc5**	**100 ± 0**	20 ± 8	27 ± 11	24 ± 6	17 ± 2	13 ± 4	14 ± 5	10 ± 4	13 ± 5
Cc5 Y1C12	14 ± 6	ND	ND	ND	ND	ND	ND	ND	ND
**Cc6**	**32 ± 7**	**100 ± 0**	24 ± 12	20 ± 4	13 ± 2	14 ± 6	12 ± 3	11 ± 4	14 ± 7
Cc6 Δ*wbuB*	ND	14 ± 7	ND	ND	ND	ND	ND	ND	ND
**Cc9**	15 ± 3	17 ± 7	**100 ± 0**	22 ± 4	17 ± 5	14 ± 5	13 ± 4	10 ± 3	14 ± 4
Cc9 Δ*wbuB*	ND	ND	20 ± 7	ND	ND	ND	ND	ND	ND
**Cc12**	19 ± 7	15 ± 5	23 ± 8	**100 ± 0**	18 ± 0	14 ± 5	15 ± 5	10 ± 3	13 ± 5
Cc12 Δ*wbtA*	ND	ND	ND	20 ± 5	ND	ND	ND	ND	ND
**Cc4**	16 ± 3	14 ± 3	**30 ± 10**	26 ± 5	**100 ± 0**	13 ± 5	13 ± 4	10 ± 2	13 ± 5
CcD3	18 ± 7	11 ± 4	18 ± 5	19 ± 3	14 ± 6	10 ± 3	12 ± 1	15 ± 4	12 ± 6
CcD5	17 ± 4	13 ± 8	16 ± 4	22 ± 4	13 ± 6	11 ± 5	13 ± 2	14 ± 6	14 ± 6
CcD6	18 ± 10	11 ± 5	20 ± 7	18 ± 3	15 ± 7	11 ± 3	14 ± 2	17 ± 4	16 ± 10
CcD10	17 ± 8	12 ± 5	18 ± 3	17 ± 2	17 ± 5	10 ± 1	14 ± 2	15 ± 6	14 ± 6
**CcD13**	16 ± 7	11 ± 5	21 ± 4	17 ± 2	12 ± 5	**99 ± 1**	12 ± 1	13 ± 4	12 ± 5
**CcD16**	18 ± 7	10 ± 4	17 ± 4	**86 ± 14**	11 ± 5	10 ± 3	13 ± 1	15 ± 5	12 ± 5
CcD18	19 ± 9	12 ± 6	17 ± 5	15 ± 2	28 ± 12	10 ± 2	15 ± 1	17 ± 6	13 ± 6
**CcD20**	17 ± 6	11 ± 6	19 ± 3	17 ± 3	**57 ± 24**	11 ± 3	12 ± 1	14 ± 4	12 ± 5
CcD25	15 ± 6	11 ± 5	18 ± 4	17 ± 2	12 ± 5	9 ± 2	11 ± 1	14 ± 4	12 ± 3
**CcD33**	20 ± 9	11 ± 6	17 ± 6	22 ± 2	13 ± 5	10 ± 3	12 ± 2	13 ± 4	**106 ± 30**
CcD34	16 ± 9	10 ± 4	16 ± 3	14 ± 2	13 ± 6	10 ± 2	12 ± 1	13 ± 4	13 ± 6
CcD35	14 ± 7	12 ± 4	15 ± 5	12 ± 1	12 ± 4	11 ± 3	12 ± 2	14 ± 3	12 ± 5
**CcD37**	15 ± 4	9 ± 3	19 ± 2	16 ± 0	12 ± 4	**100 ± 0**	11 ± 1	12 ± 4	11 ± 5
CcD39	14 ± 4	10 ± 5	18 ± 3	22 ± 2	14 ± 6	9 ± 2	11 ± 1	12 ± 3	12 ± 5
CcD40	16 ± 8	11 ± 5	19 ± 4	19 ± 4	12 ± 5	10 ± 2	13 ± 2	16 ± 5	13 ± 6
**CcD43**	20 ± 10	24 ± 14	**86 ± 5**	17 ± 1	13 ± 5	10 ± 3	12 ± 1	16 ± 1	14 ± 2
CcD44	15 ± 8	9 ± 4	25 ± 7	16 ± 0	12 ± 5	8 ± 1	11 ± 1	12 ± 4	11 ± 5
CcD47	16 ± 6	11 ± 6	17 ± 3	18 ± 0	12 ± 5	8 ± 2	12 ± 1	14 ± 3	11 ± 4
CcD51	15 ± 7	14 ± 5	18 ± 4	20 ± 4	11 ± 5	9 ± 2	12 ± 0	14 ± 4	12 ± 5
**CcD52**	16 ± 8	11 ± 7	16 ± 5	20 ± 6	11 ± 5	**83 ± 4**	13 ± 2	14 ± 4	12 ± 5
**CcD53**	19 ± 8	12 ± 6	17 ± 2	18 ± 2	12 ± 5	9 ± 2	14 ± 2	**41 ± 7**	11 ± 4
**CcD57**	17 ± 6	28 ± 18	21 ± 4	23 ± 12	**32 ± 10**	9 ± 2	13 ± 1	13 ± 4	12 ± 5
**CcD58**	18 ± 7	11 ± 5	17 ± 3	22 ± 3	**34 ± 11**	11 ± 2	14 ± 1	15 ± 5	13 ± 5
**CcD63**	15 ± 9	11 ± 5	17 ± 1	17 ± 0	29 ± 11	11 ± 5	**100 ± 0**	14 ± 5	12 ± 5
**CcD68**	**43 ± 7**	**110 ± 11**	16 ± 5	18 ± 0	13 ± 5	9 ± 2	13 ± 1	14 ± 5	11 ± 5
CcD69	14 ± 7	11 ± 6	16 ± 3	13 ± 2	12 ± 5	8 ± 3	12 ± 1	12 ± 4	11 ± 5
CcD71	15 ± 6	11 ± 5	17 ± 6	16 ± 2	13 ± 6	10 ± 2	13 ± 1	14 ± 5	13 ± 5
CcD73	19 ± 7	12 ± 7	17 ± 0	23 ± 3	15 ± 6	15 ± 7	13 ± 5	18 ± 7	13 ± 5
CcD76	13 ± 6	16 ± 8	14 ± 1	15 ± 3	15 ± 7	15 ± 7	13 ± 4	12 ± 4	13 ± 4
CcD77	14 ± 9	13 ± 4	17 ± 7	14 ± 2	15 ± 7	14 ± 6	12 ± 5	10 ± 2	13 ± 4
**CcD80**	16 ± 11	13 ± 4	19 ± 5	17 ± 3	**30 ± 9**	13 ± 5	13 ± 5	10 ± 4	13 ± 5
CcD81	16 ± 5	12 ± 4	23 ± 7	19 ± 4	22 ± 5	12 ± 5	12 ± 5	9 ± 3	11 ± 4
CcD84	17 ± 8	14 ± 7	13 ± 1	18 ± 2	29 ± 16	12 ± 5	12 ± 3	14 ± 4	13 ± 4
**CcD89**	17 ± 6	15 ± 1	20 ± 6	**95 ± 9**	14 ± 9	14 ± 5	13 ± 4	13 ± 5	13 ± 5
**CcD96**	20 ± 8	14 ± 1	19 ± 7	15 ± 1	**118 ± 37**	12 ± 4	11 ± 4	9 ± 3	13 ± 5
**CcD101**	18 ± 5	12 ± 3	13 ± 4	16 ± 1	13 ± 7	12 ± 5	10 ± 3	**100 ± 0**	12 ± 4
**CcD104**	16 ± 9	14 ± 1	18 ± 7	21 ± 4	**31 ± 11**	11 ± 4	10 ± 3	9 ± 3	11 ± 4
**CcD105**	**107 ± 28**	14 ± 2	16 ± 8	17 ± 5	14 ± 8	13 ± 5	11 ± 4	9 ± 3	12 ± 4
**CcD106**	13 ± 8	16 ± 1	21 ± 11	17 ± 2	**59 ± 24**	13 ± 5	11 ± 4	9 ± 3	13 ± 5
**CcD113**	15 ± 11	16 ± 2	19 ± 9	14 ± 0	14 ± 6	**108 ± 6**	11 ± 4	9 ± 4	12 ± 4
CcD115	15 ± 9	16 ± 2	19 ± 9	21 ± 4	16 ± 8	12 ± 4	10 ± 4	9 ± 3	12 ± 5
CcD116	14 ± 8	14 ± 2	19 ± 5	18 ± 2	15 ± 7	12 ± 5	12 ± 5	9 ± 3	13 ± 5
**CcD117**	19 ± 10	16 ± 1	19 ± 8	**99 ± 12**	14 ± 7	13 ± 6	13 ± 5	9 ± 3	13 ± 5
**CcD118**	15 ± 7	14 ± 1	20 ± 9	19 ± 4	15 ± 7	**111 ± 5**	12 ± 4	10 ± 3	12 ± 5
CcD119	15 ± 8	16 ± 2	16 ± 9	28 ± 22	15 ± 9	12 ± 4	11 ± 4	12 ± 5	12 ± 4
CcD120	15 ± 8	17 ± 3	17 ± 5	23 ± 7	16 ± 8	13 ± 5	12 ± 4	10 ± 3	12 ± 4
CcD122	12 ± 8	14 ± 1	17 ± 6	13 ± 1	14 ± 8	13 ± 5	12 ± 4	9 ± 2	12 ± 4
**CcD124**	15 ± 8	16 ± 1	19 ± 8	19 ± 4	15 ± 7	**109 ± 9**	11 ± 4	9 ± 3	12 ± 4
CcD126	13 ± 7	14 ± 1	16 ± 6	21 ± 3	15 ± 7	13 ± 6	11 ± 4	11 ± 4	12 ± 4
**CcD129**	13 ± 9	15 ± 2	21 ± 6	20 ± 2	16 ± 8	12 ± 5	14 ± 6	10 ± 2	**100 ± 0**
**CcD130**	13 ± 7	29 ± 11	**108 ± 26**	19 ± 2	11 ± 6	15 ± 6	12 ± 4	10 ± 4	13 ± 5
CcD131	12 ± 7	14 ± 2	17 ± 6	19 ± 3	14 ± 6	13 ± 6	11 ± 4	10 ± 3	12 ± 4

a Capsular serotyping was determined by ELISA on entire heat-killed bacteria. The following sera were used: Y1C12-adsorbed anti-Cc5 (A), Cc6 Δ*wbuB adsorbed anti-Cc6 (B), Cc9 ΔwbuB* adsorbed anti-Cc9 (C), Cc12 Δ*wbtA* adsorbed anti-Cc12 (D), anti-Cc4 adsorbed with all human isolates except Cc4 (E), anti CcD37 adsorbed with all human isolates (F), anti CcD63 adsorbed with all human isolates (G), anti CcD101 adsorbed with all human isolates (H), and anti CcD129 adsorbed with all human isolates (I). The readout of the ELISA was absorbance, but the results are expressed here as percentage of reactivity calculated with respect to the absorbance value obtained for the capsular type strain. Values are the mean ± standard deviation (SD) of the results from at least 3 independent experiments. Isolates with reactivities ≥30% appear in bold face. In addition, isolates with reactivities higher than 80% are highlighted in gray. ND, not determined.

### There is high capsular variability among the isolates from dog mouths.

To investigate the variability of the capsular serovars in the 44 untyped dog isolates, we generated sera against 4 randomly chosen isolates (CcD37, CcD63, CcD101, and CcD129). Since we could not generate uncapsulated mutants, because the genome sequences of these isolates are not available, the antisera were adsorbed using a mix of the 25 C. canimorsus human isolates. After validating the adsorption efficacy by immunofluorescence (Fig. S1), we screened the 52 dog isolates by ELISA (Table 1). The adsorbed anti-CcD37 serum reacted not only with CcD37 but also with CcD13, CcD52, CcD113, CcD118, and CcD124, with reactivities between 83 and 111%. All these reactions were confirmed by Western blotting (Fig. S2F). This serovar, named F, thus had a prevalence of 11.5% among dog isolates (6/52). The adsorbed anti-CcD63 reacted with the CPS of CcD63 but with no other isolate (Fig. S2G). This serovar, G, thus had a reduced prevalence of 1.9% (1/52). The adsorbed anti-CcD101 serum reacted with only one other isolate, CcD53, but this isolate did not show any CPS (Fig. S2H). This serovar, H, thus had a prevalence of 1.9% (1/52). Finally, the adsorbed anti-CcD129 serum reacted by ELISA and Western blotting with the CPS of CcD129 and CcD33 (Fig. S2I). This serovar, I, thus had a prevalence of 3.8% (2/52). There were no significant differences in the prevalences of serovars F, G, H, and I between dog and human isolates (*P* values of 0.169 for F and 1 for G, H, and I, Fisher's exact test), but while five serovars covered all 25 human isolates (100%), nine serovars covered only 18 dog isolates (34.6%) (Fig. 2). This result indicates there is a higher variability of capsular serovars among dog isolates than among human isolates.

### Detection of the capsular serovars A to E by PCR.

Our data so far clearly show that the capsular serotyping could help identify dogs hosting C. canimorsus isolates that are more virulent for humans than others. Since immunological screening methods are somehow difficult to implement in diagnostic laboratories, we tried to develop a PCR-based method using different oligonucleotide couples that would allow the identification of the 5 serovars found among human isolates.

**FIG 3:**
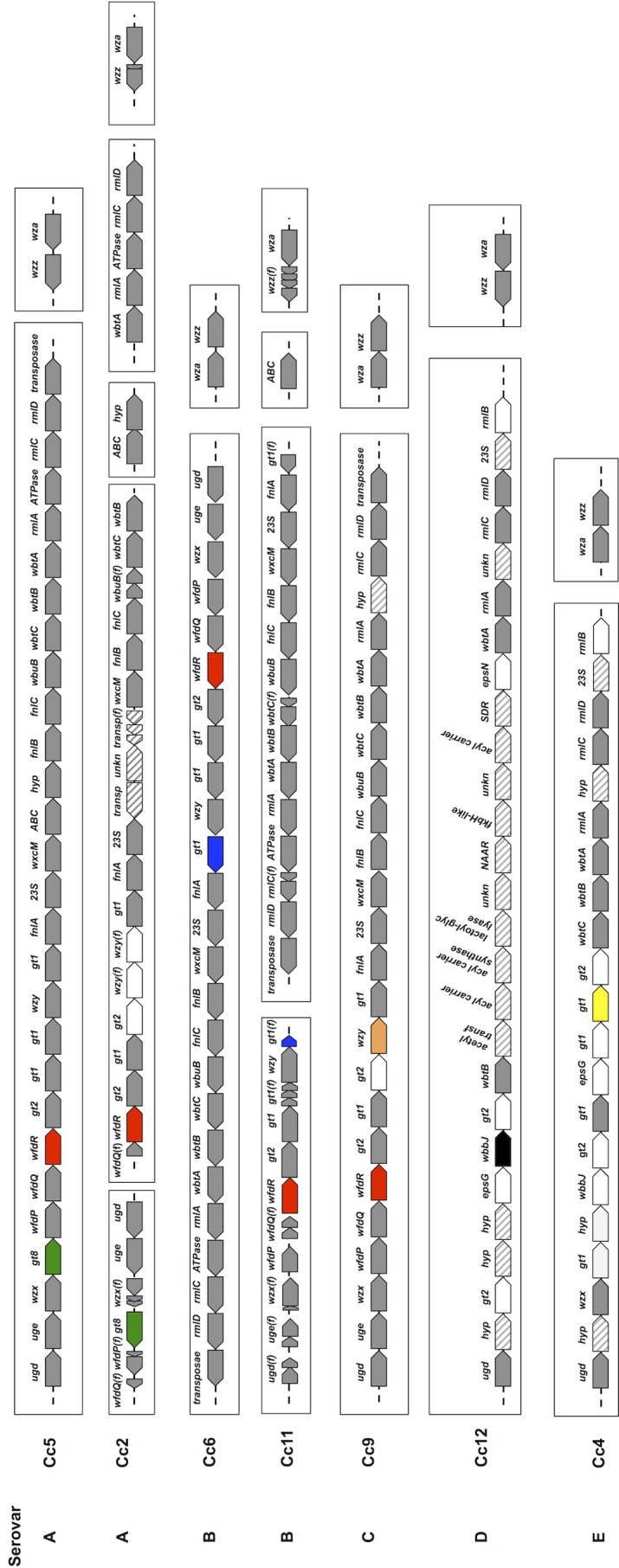
Synteny analysis of LOS/CPS loci in capsular serovars A to E. Comparison of the LOS/CPS biosynthesis and transport genetic loci of the seven C. canimorsus isolates whose genomes were sequenced. The boxes indicate different genomic loci. Homologs of the Cc5 genes are indicated in gray. The genes amplified by the A to E serovar-specific PCR are indicated in green, blue, orange, black, and yellow, respectively. The target genes amplified by the ABC serovar-specific PCR are in red. Genes indicated in white are isolate-specific genes likely involved in LOS/CPS biosynthesis. The hatched pattern indicates genes likely unrelated to LOS/CPS biosynthesis and transport. Fragmented genes are marked with *(f)*. Note that the genomes of Cc2, Cc4, Cc6, Cc9, Cc11, and Cc12 are draft genomes. For the sake of simplicity, genes are not represented to scale.

**FIG 4:**
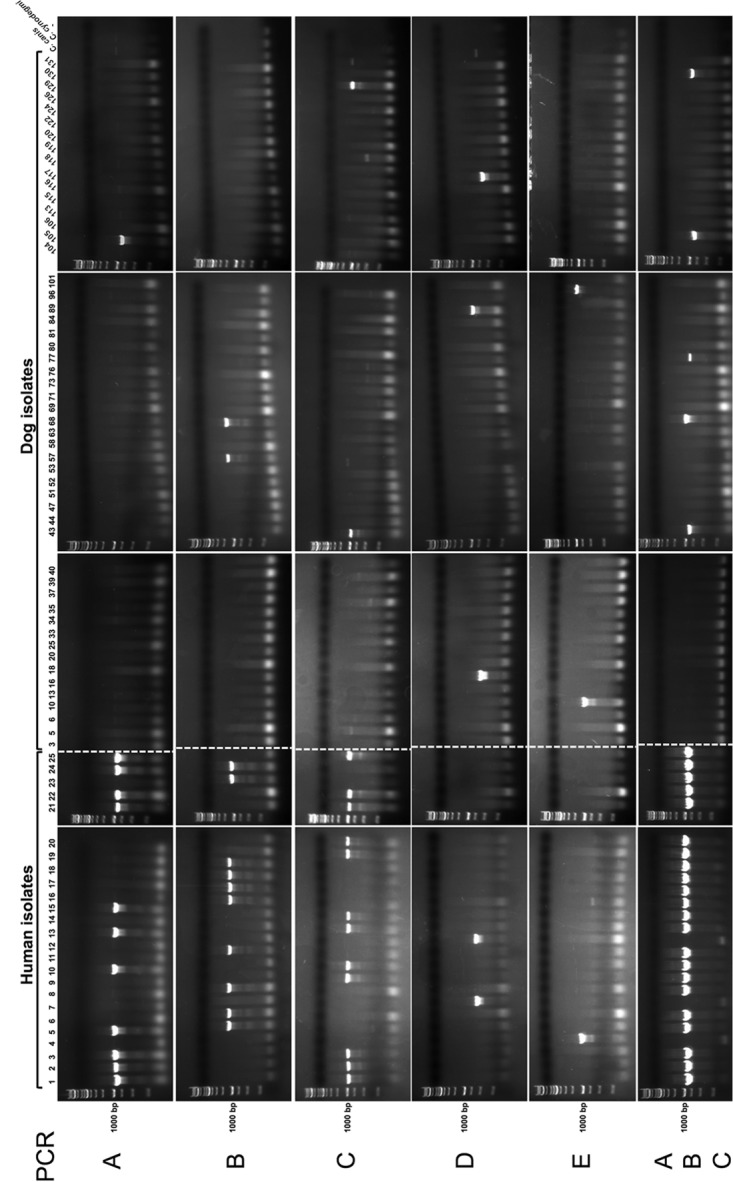
Capsular typing by PCR. PCR detection of capsular serovars A to E in C. canimorsus human and dog isolates using the oligonucleotides given in Table S3. C. canis (type strain CcD38, LMG 29146, DSM 101831) and C. cynodegmi (type strain Ccyn ATCC 49044) were used as negative controls.

We thus first compared the capsule and LOS biosynthesis loci in the seven available genomes of C. canimorsus isolates belonging to the five serovars (Cc5, Cc2, Cc6, Cc11, Cc9, Cc12, and Cc4) (Fig. 3). Looking for a gene that was specific to serovar A isolates (Cc5 and Cc2), we identified an A4GalT-like glycosyltransferase gene (*Ccan_23210* and *CCAN2_1920004* in Cc5 and Cc2, respectively) (20). Two amplimers were designed, and our complete C. canimorsus collection was tested by PCR. As shown in Fig. 4 and Table 2, this analysis detected all serovar A isolates (11 human and one dog isolate) and no other isolates.

**TABLE 2:** Summary of capsular typing of human isolates by PCR^*a*^

Strain/isolate	Results by PCR (primers)						Serovar
	A (8244 and 8245)	B (8246 and 8247)	C (8274-8275)	D (8276 and 8277)	E (8278 and 8279)	ABC (8296 and 8297)	
Cc1	X		X			X	A
Cc2	X		X			X	A
Cc3	X		X			X	A
Cc5	X	X				X	A
Cc10	X		X			X	A
Cc13	X		X			X	A
Cc15	X	X				X	A
Cc21	X		X			X	A
Cc22	X		X			X	A
Cc24	X	X				X	A
Cc25	X		X			X	A
Cc6		X				X	B
Cc8		X				X	B
Cc11		X				X	B
Cc16		X				X	B
Cc17		X				X	B
Cc18		X				X	B
Cc23		X				X	B
Cc9			X			X	C
Cc14			X			X	C
Cc19			X			X	C
Cc20			X			X	C
Cc7				X			D
Cc12				X			D
Cc4					X		E

a Positive PCR results are represented by X.

Regarding serovar B, we could not identify any gene that was unique to the Cc6 and Cc11 genomes (Fig. 3). However, while genes *CC6_1430035* and *CCAN11_10027*, both encoding a putative family 1 glycosyltransferase, were exactly conserved in Cc5 (serovar A), they were not in Cc2 (also serovar A). Aligning *CC6_1430035* and *CCAN11_10027* with their homologs from Cc2 (*CCAN2_1430008*) and Cc9 (serovar C) (*CCAN9_740032*) (20) revealed a difference in the 16 bp immediately downstream of the start codon (Fig. S3). Since the two serovar B isolates (Cc6 and Cc11) had the exact same gene sequence, shared by only one of the two serovar A isolates (Cc5), we tested whether the exact same gene sequence would not be shared by all serovar B isolates. We thus designed two oligonucleotides to amplify this specific gene region and, as shown in Fig. 4 and Table 2, by this PCR, we could indeed detect all 7 serovar B human isolates, as well as the only serovar B dog isolate (CcD68). As expected, we could also detect Cc5, as well as two other serovar A isolates, namely, Cc15 and Cc24. Surprisingly, the PCR gave a positive result for one dog isolate (CcD57) that did not belong to any of the 5 serovars (Table 1 and Fig. S2E and S4A) and thus represents a false positive. Nevertheless, with this PCR, we could detect all serovar B isolates of our collection, and this analysis, if combined with the one specific for serovar A, allowed the discrimination between serovars A and B. Indeed, serovar B isolates were positive for PCR B but negative for PCR A.

Regarding serovar C, we could not identify any gene unique to the Cc9 genome, but *CCAN9_740031*, encoding a putative O-antigen polymerase (*wzy*), had a homolog only in one serovar A isolate, Cc2 (Fig. 3). We thus tested by PCR whether this gene would be shared by all serovar C isolates. As shown in Fig. 4 and Table 2, we could detect all serovar C isolates, namely, the 4 from humans (Cc9, Cc14, Cc19, and Cc20), as well as the two from dogs (CcD43 and CcD130). This PCR thus allows the detection of the serovar C isolates and, if combined with the PCR for serovar A, it allows discrimination between these two serovars. Indeed, serovar C isolates are positive for PCR C but negative for PCR A.

Concerning serovar D, the Cc12 LOS/CPS locus was previously shown to be very divergent from the ones of serovar A, B, and C isolates, with a limited number of conserved genes (20) (Fig. 3). We chose to amplify gene *CCAN12_760043* encoding a putative lipopolysaccharide biosynthesis *O*-acetyltransferase (WbbJ) that had no homologs in the loci of all the other serovars. As shown in Fig. 4 and Table 2, this PCR exclusively detected the serovar D isolates, and it detected them all (Cc12, Cc7, CcD16, CcD89, and CcD117).

Finally, as for Cc12, the serovar E strain Cc4 LPS/CPS locus strongly diverged from those of all the other serovars (Fig. 3). We thus chose as the target gene a Cc4 unique gene, namely, *CC4_530066*, encoding a glycosyltransferase 1 family protein. As shown in Fig. 4 and Table 2, this PCR detected the Cc4 and CcD96 serovar E isolates. Among the other isolates, only CcD10 gave a positive result, although it did not react with the E antiserum (Table 1 and Fig. S4B) and could thus be considered a false positive. In summary, in order to determine the serovar of a C. canimorsus isolate, the five (A to E) PCRs should be performed and the results interpreted as follows: (i) all isolates that are positive for PCR A belong to serovar A, (ii) isolates that are positive for PCR B belong to serovar B if they are not positive for PCR A, (iii) isolates that are positive for PCR C are serovar C if they are not positive for PCR A, (iv) isolates that are positive for PCR D are serovar D, and (v) isolates that are positive for PCR E are serovar E (Tables 2 and 3).

**TABLE 3:** Interpretation of PCR typing results^*a*^

Serovar	Results by PCR (primers)					
	A (8244 and 8245)	B (8246 and 8247)	C (8274-8275)	D (8276 and 8277)	E (8278 and 8279)	ABC (8296 and 8297)
A	X					X
	X	X				X
	X		X			X
B		X				X
C			X			X
D				X		
E					X	

a Positive PCR results are represented by X.

In conclusion, capsular serotyping can be done by PCR (Tables 2 and 3), with a very limited margin of error (2 false-positive dog isolates).

Next, given the higher prevalences of serovars A, B, and C (22/25) among human isolates, we decided to develop a PCR that would allow the detection of all serovar A, B, and C isolates. To this aim, taking advantage of the high similarity among the LOS/CPS loci of the isolates belonging to serovars A, B, and C, we designed two amplimers specific to the conserved region of the putative glycosyltransferase *wfdR* ortholog genes of serovar A (*Ccan_23240* in Cc5 and *CCAN2_1430002* in Cc2), serovar B (*CC6_1430040* in Cc6 and *CCAN11_2010013* in Cc11), and serovar C (*CCAN9_740027*). As shown in Fig. 4 and Table 2, by this PCR, we could detect all isolates belonging to serovars A, B, and C. Among the non-A, -B, or -C isolates, only CcD77 gave an amplification but not of the same size (Fig. 4). This PCR, allowing the fast identification and, specifically, all the C. canimorsus isolates belonging to serovar A, B, or C (Table 3), could thus be a valuable tool in terms of prevention.

## DISCUSSION

Here, we show that all 25 out of 25 C. canimorsus isolates from human infections and 18 dog isolates out of 18 tested are endowed with a CPS. We thus confirm our previous observation, where a capsular-like polysaccharide structure was found in 10 human isolates (20). This result further reinforces the commonality of the presence of a CPS in C. canimorsus. In addition, we developed a serotyping scheme based on the capsular antigens, and we described nine serovars (A to I). LOS and CPS syntheses are genetically linked in strain Cc5, resulting in similar polysaccharide unit compositions in the two structures (20). For serovars B to I, we also found shared epitopes between the CPS and LOS (data not shown). Even more, the antiserum directed against the CPS/LOS from serovar C recognized the LOS but not the CPS from some serovar A isolates (data not shown), revealing some complexity in the CPS/LOS relationship. Because of this complexity and because it is the CPS rather than the LOS that impacts the host-pathogen interaction (20), we based our typing scheme on the CPS only. However, because of this cross-reaction, the distinction between serovars A and C must be done by Western blotting and not by immunofluorescence or ELISA. Because Western blotting is a tedious technique for clinical laboratories, we set up a PCR method for capsular serotyping. The cross-reaction between the LOS of serovar A and some isolates of serovar C also appeared when the typing was done by PCR, but combining the two PCRs allows a determination of the serovar without any ambiguity. Further work will be required to understand the molecular mechanisms underlying these LOS cross-reactions, but carbohydrate chemistry always represents a long-term project.

The nine serovars described covered only 18 dog isolates out of 52 isolates tested, while five serovars covered only the 25 human isolates. Thus, there was a high variety of capsular serovars among the dog isolates. In contrast, only three serovars (A, B, and C) covered 88% of the human isolates tested (22/25), while they covered only 4 dog isolates (7.7%). There was thus a very strong enrichment of serovars A, B, and, to a lesser extent, C, in human isolates compared to dog ones. Interestingly, these three dominant capsular serovars were not restricted to a geographical area but were rather distributed worldwide. This observation clearly indicates that the strains belonging to serovars A, B, and, possibly C are more virulent for humans than strains from the other serovars. This sets the basis for the prevention of these severe infections. To this aim, one could envision the detection of potentially more dangerous dogs using a PCR carried out directly on the dog's saliva and simultaneous monitoring of the three more virulent serovars. Our results on collection isolates have indeed shown that PCR is reliable, with a very limited number of false positives and, in our experience, no false negatives. Owners of a dog hosting a serovar A, B, or C strain should be educated to limit the contact with the dog's saliva, and if a bite or a lick occurs, to apply strict hygiene measures. In addition, splenectomized and more generally immunocompromised persons should not consider adopting a dog hosting a virulent C. canimorsus strain.

Ideally, more human isolates should be serotyped to reinforce the correlation between some capsular serovars and human infections, but their collection is very tedious due to the rarity of the disease and the fastidious character of these bacteria.

There was no significant difference in the distributions of capsular serovars D and E among dog (4/52) and human (3/25) isolates, which suggests that they are probably not more virulent than most dog strains. This observation leads to the conclusion that, while a majority of the patients (88% of our sample) are infected with virulent strains (serovars A, B, possibly C), a minority of patients (12% of our sample) could have been infected by strains that belong to a less-virulent serovar (D and E). This is consistent with the fact that some patients were obviously at risk while others had no history of immunodeficiency. In agreement with this hypothesis, the patient infected with Cc4 (serovar E) was highly immunocompromised (27), and the patient infected with Cc12 (serovar D) was splenectomized (2) (Table S1). Hence, splenectomized and more generally immunocompromised persons should be extremely cautious when interacting with dogs hosting C. canimorsus, regardless of the serovar of the C. canimorsus strain hosted.

It is likely that it is the capsule itself that confers enhanced virulence to serovars A, B, and C, as is classical for other pathogens (21). In support of this, the capsule of the type strain Cc5 has been recently shown to provide resistance to phagocytosis by macrophages, to killing by 10% human serum, and to killing by the cationic antimicrobial peptide polymyxin B (20). These results suggest that the serovar A CPS might indeed participate in the innate immune evasion in humans. Ideally, these *in vitro* data should be reinforced by *in vivo* studies, but the lack of a relevant sepsis animal model to study C. canimorsus infections prevents such confirmation. Further *in vitro* work could determine if the capsular serovars A, B, and C provide the strains with higher resistance to the innate immune system. However, we cannot exclude that other virulence factors could be genetically linked to some capsular serovars. It would thus be interesting to compare the whole genomes looking for genes that would be shared in serovar A, B, and C strains and absent in serovar F, G, H, and I strains.

## MATERIALS AND METHODS

### Bacterial strains, isolates, and culture conditions.

The bacterial strains and isolates used in this study are listed in the Tables S1 and S2. C. canimorsus was grown on heart infusion agar (HIA; BD Difco, Franklin Lakes, NJ, USA) supplemented with 5% sheep blood (SB; Oxoid, Basingstoke, UK) plates for 48 h at 37°C with 5% CO_2_. Escherichia coli was routinely grown in lysogeny broth (LB; Invitrogen, Waltham, MA, USA) at 37°C. Antibiotics used as selective agents were added at the following concentrations: 100 μg/ml ampicillin (AMP) and 50 μg/ml kanamycin (KAN) for E. coli and 20 μg/ml gentamicin (GEN), 10 μg/ml erythromycin (ERY), and 10 μg/ml cefoxitin (FOX) for C. canimorsus. Unless otherwise stated, products were purchased from Sigma-Aldrich (Darmstadt, Germany).

### Antiserum production and adsorption.

Bacteria were grown for 2 days on SB plates supplemented with GEN, gently scraped from the agar, resuspended, and washed in phosphate-buffered saline (PBS). Bacteria were fixed overnight in 0.3% paraformaldehyde (PFA), washed in PBS, and inoculated to a rabbit to generate polyclonal sera. Sera were generated at the University of Namur (Belgium) or at the Centre d'Économie Rurale (CER Groupe, Aye, Belgium). The respective animal welfare committees approved the animal handling and procedures. Polyclonal sera were adsorbed by incubation with an excess of PFA-fixed noncapsulated mutant bacteria, unless stated otherwise in the Results. Incubations were done on a rotating wheel at room temperature (RT) and repeated four times. Bacteria were removed by repeated centrifugations. Adsorption efficacy was assessed by immunofluorescence as follows. Glass coverslips were coated with poly-d-lysine (10 μg/ml in PBS for 1 h at 37°C), washed, and incubated for 30 min at 37°C with 300 μl of a bacterial suspension adjusted to an optical density at 600 nm (OD_600_) of 0.25. Coverslips were then washed, and bacteria were fixed for 15 min with 4% PFA. Coverslips were washed again and blocked with 1% bovine serum albumin (BSA) for 1 h at RT. Bacteria were stained with the adsorbed sera (1:1,000 in PBS) for 1 h at RT, followed by incubation with an Alexa Fluor 488-coupled donkey anti-rabbit antibody (1:5,000 in PBS; Life Technologies, Waltham, MA, USA) or a Texas Red coupled goat anti-rabbit antibody (1:1,000 in PBS; SouthernBiotech, Birmingham, AL, USA) for 45 min. Coverslips were mounted using Mowiol mounting medium, and images were acquired with an Axio Imager.Z1 (Zeiss, Oberkochen, Germany) and analyzed using the Zen 2012 software (Zeiss).

### Mutagenesis by allelic exchange.

Mutagenesis of the Cc6, Cc9, and Cc12 strains was performed as previously described (28). The C. canimorsus deletion mutants and the E. coli strains used are listed in the Table S2. Briefly, replacement cassettes with flanking regions spanning approximately 500 bp homologous to regions directly framing targeted genes were constructed with a three-fragment overlapping PCR strategy. First, two PCRs were performed on 100 ng of Cc6, Cc9, or Cc12 genomic DNA with primers 1.1 and 1.2 for the upstream flanking regions and with primers 2.1 and 2.2 for the downstream regions (Table S3). Primers 1.2 and 2.1 contained an additional 5′ 20-nucleotide extension homologous to the *ermF* insertion cassette. The *ermF* resistance cassettes were amplified from plasmid pMM13 (28) DNA, with primers 3.1 and 3.2. All three PCR products were cleaned and then mixed in equal amounts for PCR using Phusion polymerase (Finnzymes, Espoo, Finland). The initial denaturation was at 98°C for 2 min, followed by 12 cycles without primers to allow annealing and elongation of the overlapping fragments (1 cycle consists of 98°C for 30 s, 50°C for 40 s, and 72°C for 2 min). After the addition of external primers (primers 1.1 and 2.2), the program was continued with 20 cycles (1 cycle consists of 98°C for 30 s, 50°C for 40 s, and 72°C for 2 min 30 s) and, finally, 10 min at 72°C. The final PCR products consisting of *locus*::*ermF* insertion cassettes were then digested with PstI and SpeI (New England BioLabs, Ipswich, MA, USA) for cloning into the appropriate sites of the C. canimorsus suicide vector pMM25 (28). The resulting plasmids were transferred by RP4-mediated conjugative DNA transfer from E. coli S17-1 to the corresponding C. canimorsus strains to allow integration of the insertion cassette. Transconjugants were then selected for the presence of the *ermF* cassette on erythromycin-containing plates and checked for sensitivity to cefoxitin. Deletion of the appropriate regions was verified by PCR.

### Western blotting of polysaccharide structures.

Bacteria were harvested by gently scraping colonies off the agar surface of a GEN SB plate and resuspended in PBS. Bacterial suspensions were adjusted to an OD_600_ of 1 in PBS. Seven hundred fifty microliters of the suspension was pelleted and resuspended in 125 μl of loading buffer (1% sodium dodecyl sulfate [SDS], 10% glycerol, 50 mM dithiothreitol, 0.02% bromophenol blue, 45 mM Tris [pH 6.8]). Samples were heated for 10 min at 99°C. Proteinase K (VWR Chemicals, Radnor, PA, USA) was added to a final concentration of 50 μg/ml, and samples were incubated overnight at 37°C. Subsequently, samples were heated for 10 min at 99°C, and proteinase K was added again at the same final concentration. Samples were incubated for 3 h at 55°C, heated for 5 min at 99°C, and loaded on a 12% polyacrylamide gel. After SDS-PAGE, proteinase K-resistant structures were transferred onto a nitrocellulose membrane (GE Healthcare, Chicago, IL, USA). Membranes were blocked and incubated with polyclonal crude or adsorbed sera (dilutions ranging from 1:400 to 1:8,000), followed by incubation with a horseradish peroxidase (HRP)-coupled goat anti-rabbit polyclonal antibody (1:2,000; Dako Agilent Technologies, Santa Clara, CA, USA). The membranes were revealed using a chemiluminescent substrate (KLP, Gaithersburg, MD, USA) on an Amersham Imager 600 (GE Healthcare). Blocking and all incubations were conducted in 5% nonfat dry milk diluted in PBS–0.05% Tween.

### Capsular serotyping by ELISA.

Bacterial suspensions were adjusted to an OD_600_ of 0.5 and were killed by an incubation of 30 min at 70°C. The heat-killed bacterial suspensions were used to coat 96-well plates (Thermo Scientific, Waltham, MA, USA) overnight at 4°C. The next day, the plates were washed to remove unfixed bacteria and were blocked for 1 h at RT with 1% BSA in PBS. The plates were washed and incubated with adsorbed polyclonal serum (1:1,000 to 1:1,500 in PBS) for 1 h at RT. The plates were washed again and incubated with HRP-coupled goat anti-rabbit polyclonal antibody for 1 h at RT (1:2,000 in PBS; Dako Agilent Technologies). The plates were then washed and revealed using 3,3′,5,5′-tetramethylbenzidine (TMB) as a chromogenic substrate.

### Capsular serotyping by PCR.

Bacteria were grown on SB plates supplemented with GEN, and a single colony was resuspended in 100 μl of double-distilled water (ddH_2_O) and heated for 15 min at 98°C. Two microliters were used as the template for amplification. PCR detection was performed using the Promega Go *Taq* G2 polymerase (Madison, WI, USA) under the following conditions: initial denaturation at 95°C for 4 min, followed by 35 cycles of denaturation at 95°C for 30 s, annealing at 52°C for 45 s, extension at 72°C for 1 min and 30 s, and a final extension at 72°C for 7 min.

### Synteny analysis.

Synteny statistics were obtained using the MicroScope PkGDB synteny statistics tool (https://www.genoscope.cns.fr/agc/microscope/home/index.php) (29). Putative orthologous relations based on the bidirectional best-hit (BBH) criterion were considered for at least 35% sequence identity on 80% of the length of the smallest protein. For the synteny analysis, all possible kinds of chromosomal rearrangements are allowed (inversion, insertion/deletion), and the gap parameter, representing the maximum number of consecutive genes which are not involved in a synteny group, is set to five genes.

### Statistical analysis.

Statistical significance was evaluated by Fisher's exact tests using the BiostaTGV website (https://marne.u707.jussieu.fr/biostatgv).

### Accession number(s).

Sequences for genes used in this study have been deposited in the European Nucleotide Archive under accession numbers LT838810, LT838811, and LT838812 and in GenBank under accession numbers NC_015846.1, CDOJ01000104.1, CDOJ01000050.1, CDOK01000001.1, CDOK01000115.1, and CDOE01000074.1 (see Table S4 in the supplemental material).

## Supplementary Material

Supplemental material
