# Supplementary material for: Identification of Virulent Capnocytophaga canimorsus Isolates by Capsular Typing
Source: J Clin Microbiol. 2017 May 23;55(6):1902–14. doi: 10.1128/JCM.00249-17 (PMC5442547; doi:10.1128/JCM.00249-17)
Supplement: Supplemental material [file supp_55_6_1902__index.html]

Identification of Virulent Capnocytophaga canimorsus Isolates by Capsular Typing — Supplemental material 

# Identification of Virulent Capnocytophaga canimorsus Isolates by Capsular Typing

## Supplemental material

- Supplemental file 1 -

  Fig. S1 (Control of serum adsorption efficacy by immunofluorescence microscopy), S2 (Analysis of high-molecular-weight polysaccharide structures from dog isolates found positive by ELISA screening), S3 (Detection of serovar B by PCR), and S4 (Analysis of high-molecular-weight polysaccharide structures from dog isolates found positive by PCR screening) and Tables S1 (*C. canimorsus* isolates from patients and dogs used in this study), S2 (*C. canimorsus* mutants and *E. coli* strains used in this study), S3 (Oligonucleotides used in this study), S4 (Accession numbers of genes used in this study), and S5 (Capsular serovar distribution according to isolate geographical origin)

  PDF, 5.0M
