# Supplementary material for: Identification of Virulent Capnocytophaga canimorsus Isolates by Capsular Typing
Source: J Clin Microbiol. 2017 May 23;55(6):1902–14. doi: 10.1128/JCM.00249-17 (PMC5442547; doi:10.1128/JCM.00249-17)
Supplement: Supplemental material [file JCM.00249-17_zjm999095527s1.pdf]

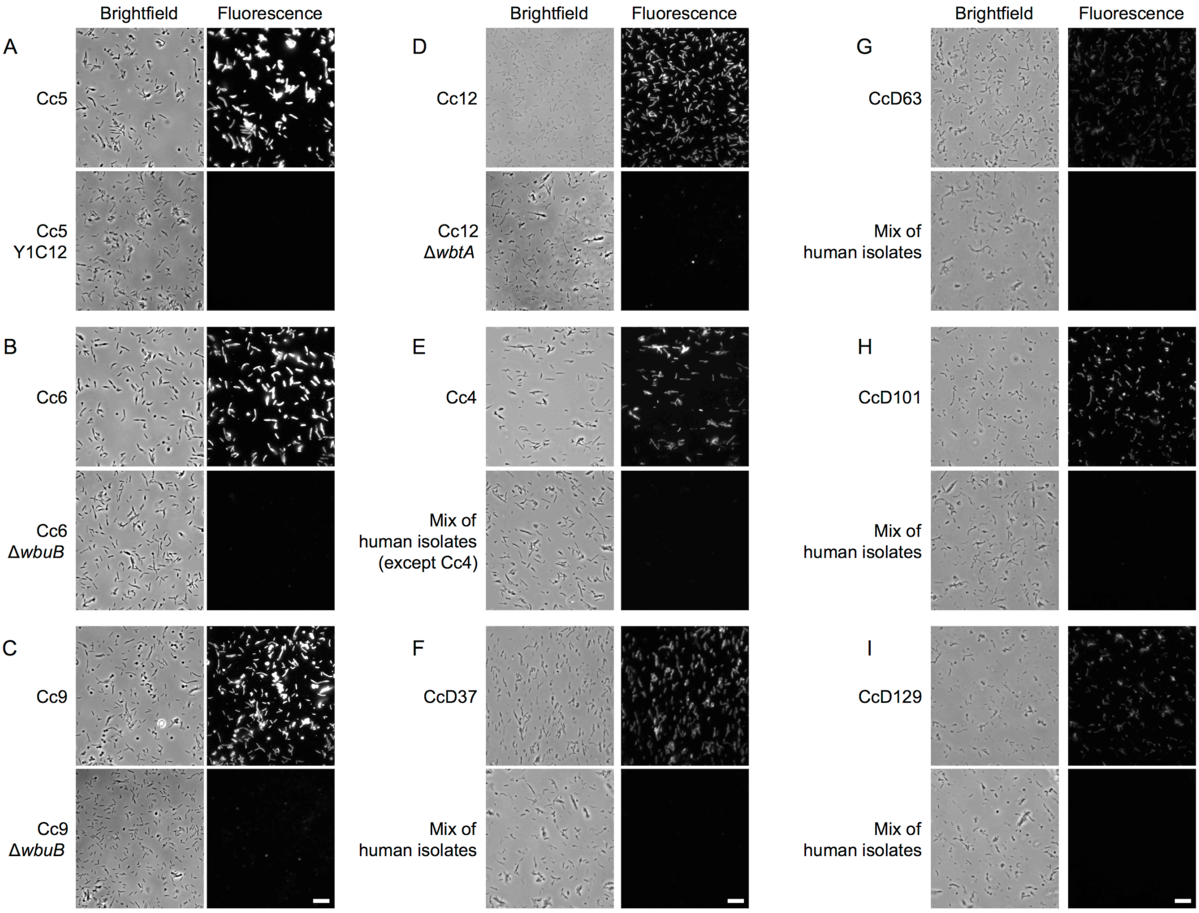

Figure S1. Control of the serum adsorption efficacy by immunofluorescence microscopy. Paraformaldehyde-fixed bacteria were stained using the following sera: Y1C12-adsorbed anti-Cc5 (A), Cc6 Δ*wbuB*-adsorbed anti-Cc6 (B), Cc9 Δ*wbuB*-adsorbed anti-Cc9 (C), Cc12 Δ*wbtA*-adsorbed anti-Cc12, anti-Cc4 adsorbed with all human isolates except Cc4 (E), anti-CcD37 adsorbed with all human isolates (F), anti-CcD63 adsorbed with all human isolates (G), anti-CcD101 adsorbed with all human isolates (H), and anti CcD129 adsorbed with all human isolates used in this study (I). Bar, 10 µm.

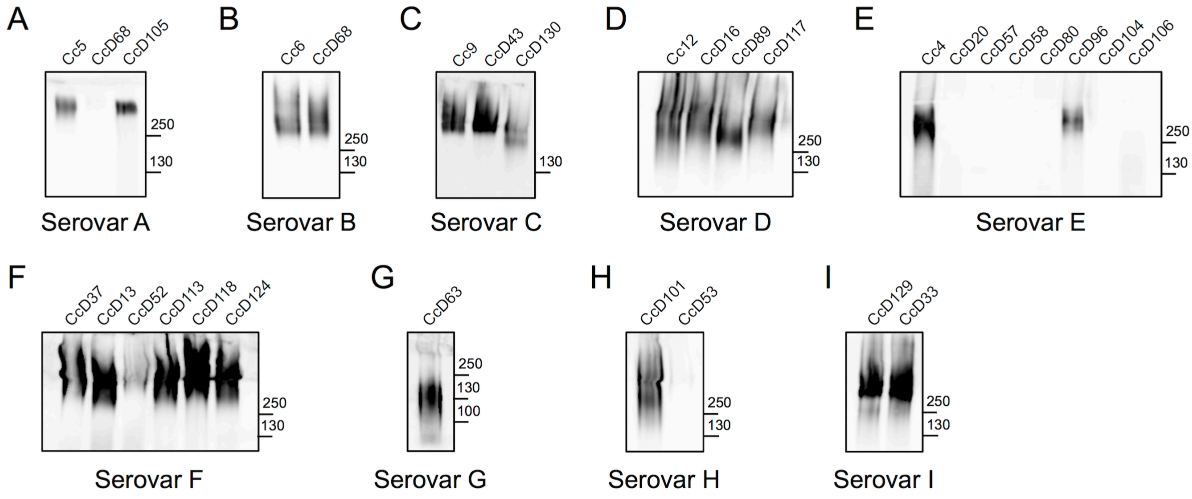

Figure S2. Analysis of high molecular weight polysaccharide structures from the dog isolates found positive by ELISA screening. Western blot analysis of proteinase K treated lysates of *C. canimorsus* dog isolates using the following sera: Y1C12 adsorbed anti-Cc5 (A), Cc6 Δ*wbuB* adsorbed anti-Cc6 (B), Cc9 Δ*wbuB* adsorbed anti-Cc9 (C), Cc12 Δ*wbtA* adsorbed anti-Cc12 (D), anti-Cc4 adsorbed with all human isolates except Cc4 (E), anti CcD37 adsorbed with all human isolates (F), anti CcD63 adsorbed with all human isolates (G), anti CcD101 adsorbed with all human isolates (H), and anti CcD129 adsorbed with all human isolates (I). Numbers correspond to molecular mass markers in kDa.

Serovar	Isolate	Locus	Sequence
B	Cc6	(*CC6_1430035*)	ATGATTAACAAAATTCTAATAGATAATTCAAATCTTTTTGCA…
B	Cc11	(*CCAN11_10027*)	ATGATTAACAAAATTCTAATAGATAATTCAAATCTTTTTGCA…
A	Cc5	(*Ccan_23290*)	ATGATTAACAAAATTCTAATAGATAATTCAAATCTTTTTGCA…
A	Cc2	(*CCAN2_1430008*)	ATG – – – – AAAATATTAATAGATAATTCAAACCTTTTTGCA…
C	Cc9	(*CCAN9_740032*)	ATG – – – – AAAATATTAATAGATAATTCAAACCTTTTTGCA…

Figure S3. Detection of the serovar B by PCR. Alignment of the first 42 nucleotides of the glycosyl transferase 1 genes amplified for the detection of the serovar B. Underlined is the sequence of the oligonucleotide (8246) that anneals to the target genes. Dashed lines indicate missing nucleotides while in red are the mismatches.

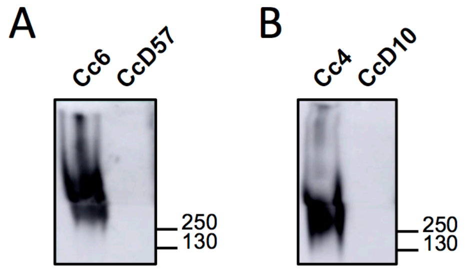

Figure S4. Analysis of high molecular weight polysaccharide structures from the dog isolates found positive by PCR screening. Western blot analysis of proteinase-K treated lysates of the dog isolates: CcD57 using the Cc6 Δ*wbuB* adsorbed anti-Cc6 serum (A) and CcD10 using anti-Cc4 adsorbed with all human isolates except Cc4 serum (B). Cc4 and Cc6 proteinase-K treated lysates have been loaded as controls. Numbers correspond to molecular mass markers in kDa.

Table S1: *C. canimorsus* isolated from patients and dogs used in this study

Clinical strains/isolates						
**Strain/ Isolate ID**	**Infection outcome**	**Patient medical history**	**Collection**	**History and geographical origin**	**Year of isolation**	**Reference**
Cc1	NK	NK	BCCM/LMG 11511; CCUG 17234; strain P810; strain SSI P810	BCCM/LMG < CCUG Sweden < W.Frederiksen < J.Ursing, Malmö, Sweden	NK	(1)
Cc2	Fatal	47 y/o F, previously healthy	…	G. Wauters & M. Delmee, Cliniques Universitaires St Luc, Brussels, Belgium	1989	(2)
Cc3	Fatal	Elderly, chronic respiratory disease and alcohol abuse	…	G. Wauters & M. Delmee < Sint-Jan Hospital, Brugges, Belgium	1990	(3)
Cc4	Fatal	49 y/o F, HIV-HCV coinfection, blindness due to CMV retinitis, aortic valve and ascending aorta replacement	…	J. Schrenzel, Hopitaux Universitaires de Genève, Switzerland	2010	(4)
Cc5	Fatal	NK	BCCM/LMG 28512	G. Wauters & M. Delmee < Clinic of Libramont, Libramont, Belgium	1995	(5)
Cc6	NK	NK	…	KU Leuven, Leuven, Belgium	1996	(6)
Cc7	NK	NK	…	G. Wauters & M. Delmee, < KU Leuven, Leuven, Belgium	1998	(5)
Cc8	NK	52 y/o M, previously healthy	…	M. Delmee < Liège, Belgium	2004	(6)
Cc9	NK	NK	BCCM/LMG 11510, CCUG 12569, CDC A3626	BCCM/LMG, CCUG < R. Weaver, CDC, Atlanta, Georgia < Virginia, USA	1965	(7)
Cc10	NK	NK	BCCM/G 11541, CCUG 24741, ATCC 35978, CDC C8936	BCCM/LMG, MCCM, ATCC < R. Weaver, CDC, Atlanta, Georgia < California Health Dept., California, USA	NK	(7)

Cc11	NK	NK	BCCM/LMG 11551, MCCM 01373	BCCM/LM < MCCM < A. von Graevenitz, Unersität Zurich, Switzerland	1990	(7)
Cc12	Recovery	17 y/o M, splenectomized	ATCC 35979, CDC 7120, CCUG 53895	ATCC < R.Weaver, CDC Atlanta Georgia < California Health Dept. < San Antonio Community Hospital, California, USA	1961	(8)
Cc13	Fatal	54 y/o M, previously healthy	…	F.S. Stals, Laurentius Ziekenhuis, Roermond, The Netherlands	2008	(9)
Cc14	NK	NK	…	R. Jarsumbeck, Medizinisches labor Ostsachsen, Dresden, Germany	2011	(6)
Cc15	Fatal	NK	…	K. Mühlemann, University Hospital Bern, Switzerland	2011	(6)
Cc16	Fatal	59 y/o M, colon cancer, intestinal occlusion	…	G. Glupczynski, Centre Hospitalier Universitaire Mont Godinne < D. Olivier, Hopital Univ. Erasme, Brussels, Belgium	NK	(6)
Cc17	Recovery	77 y/o F, no immunosuppression known	…	G. Glupczynski, Centre Hospitalier Universitaire Mont Godinne < D. Olivier, Hopital Univ. Erasme, Brussels, Belgium	NK	(6)
Cc18	Recovery	62 y/o F, kidney transplantation	…	G. Glupczynski, Centre Hospitalier Universitaire Mont Godinne < D. Olivier, Hopital Univ. Erasme, Brussels, Belgium	NK	(6)
Cc19	Recovery	49 y/o M, cardiovascular history	…	A. Magnette, Centre Hospitalier Universitaire Mont-Godinne < M Delmée < Clinique Saint Pierre, Ottignies, Belgium	2010	(6)
Cc20	NK	NK	CCUG 55909	CCUG < E. Ek, Blood Dept, PHLS, Göteborg, Sweden < UK National External Quality assessment, Colindale, London, UK	2008	…
Cc21	NK	45 y/o M	CCUG 60839	CCUG < E. Ek, Blood Dept, PHLS, Göteborg, Sweden	2011	…
Cc22	NK	NK	CCUG 20318	CCUG < W. Frederiksen, Statens Seruminstitut, Copenhagen, Denmark	1986	…

Cc23	NK	NK	CCUG 48899	CCUG < V. Roux & D. Raoult, Marseille, France	…	…
Cc24	NK	56 y/o M	CCUG 67384	CCUG < PHLS, Uddevalla < Trollhätten, Sweden	2015	…
Cc25	NK	58 y/o M	CCUG 66222	CCUG < I. Adlerberth, Blood Dept, PHLS, Sahlgrenska University Hospital, Göteborg, Sweden	2014	…

**Dog isolates**					
**Strain ID**	**Biological history**	**Collection**	**Geographical history**	**Year of isolation**	**Reference**
CcD3-CcD101	Dog mouth	…	Switzerland	2008	(6)
CcD104-CcD105	Dog mouth	…	Switzerland	2005	(6)
CcD106	Dog mouth	…	Switzerland	2006	(6)
CcD113-CcD131	Dog mouth	…	Belgium	2014	(6)

All human isolates / strains listed were isolated from human septicemia cases except Cc4, which was isolated from a prosthetic aortitis case.

NK, not known by the authors. F, female; M, male. y/o, year old. HIV, human immunodeficiency virus; HCV, hepatitis C virus; CMV, cytomegalovirus. BCCM/LMG, belgian co-ordinated collections of microorganisms / laboratory of microbiology University of Ghent. CCUG, culture collection, University of Göteborg, Sweden. MCCM, medical culture collection Marburg. PHLS, public health laboratory service.

References

1. Heltberg O, Busk HE, Bremmelgaard A, Kristiansen JE, Frederiksen W. 1984. The cultivation and rapid enzyme identification of DF-2. Eur J Clin Microbiol 3:241-243.
2. Hantson P, Gautier PE, Vekemans MC, Fievez P, Evrard P, Wauters G, Mahieu P. 1991. Fatal Capnocytophaga canimorsus septicemia in a previously healthy woman. Ann Emerg Med 20:93-94.

3. Vanhonsebrouck AY, Gordts B, Wauters G, Van Landuyt HW. 1991. Fatal septicemia with Capnocytophaga canimorsus in a compromised host. A case report with review of the literature. Acta Clin Belg 46:364-370.
4. Rougemont M, Ratib O, Wintsch J, Schrenzel J, Hirschel B. 2013. Capnocytophaga canimorsus prosthetic aortitis in an HIV-positive woman. J Clin Microbiol 51:2769-2771.
5. Shin H, Mally M, Kuhn M, Paroz C, Cornelis GR. 2007. Escape from immune surveillance by Capnocytophaga canimorsus. J Infect Dis 195:375-386.
6. Renzi F, Dol M, Raymackers A, Manfredi P, Cornelis GR. 2016. Only a subset of C. canimorsus strains is dangerous for humans. Emerg Microbes Infect 5:e29.
7. Vandamme P, Vancanneyt M, van Belkum A, Segers P, Quint WG, Kersters K, Paster BJ, Dewhirst FE. 1996. Polyphasic analysis of strains of the genus Capnocytophaga and Centers for Disease Control group DF-3. Int J Syst Bacteriol 46:782-791.
8. Butler T, Weaver RE, Ramani TK, Uyeda CT, Bobo RA, Ryu JS, Kohler RB. 1977. Unidentified gram-negative rod infection. A new disease of man. Ann Intern Med 86:1-5.
9. Kleijnen-Grebien B, Boorsma S, Stals FS, van Schelven R. 2008. Fatal case of sepsis with Capnocytophaga canimorsus after a minor dog bite. Ned Tijdschr Geneeskd 152:1882-1885.

Table S2: *C. canimorsus* mutants and *E. coli* strains used in this study

Strain ID	Genotype	Reference
E. coli		
Top10	F^- *mcrA* Δ(*mrr-hsdRMS-mcrBC*) ϕ80*lacZ*ΔM15 Δ*lacX*74 *recA1 araD139* Δ(*araleu*)*7697 galU galK rpsL endA1 nupG*; Sm^r	Invitrogen
S17-1	*hsdR17 recA1* RP4-2-*tet::*Mu1*kan::*Tn7; Sm^r	(1)
C. canimorsus		
Y1C12	*C. canimorsus* 5 *Ccan_23370*::Tn*4351*; Ery^r	(2)
Cc6 Δ*wbuB*	Replacement of *Cc6_1430029* in *C. canimorsus* 6 by *ermF* using primers 7958, 7959, 7962, 7975, 7961, 7974; Ery^r	This study
Cc9 Δ*wbuB*	Replacement of *CCAN9_740038* in *C. canimorsus* 9 by *ermF* using primers 7958, 7959, 7960, 7961, 7962, 7963; Ery^r	This study
Cc12 Δ*wbtA*	Replacement of *CCAN12_760057* in *C. canimorsus* 12 by *ermF* using primers 8063, 8064, 8065, 8066, 8067, 8068; Ery^r	This study

References

1. Simon R, Priefer U, Puhler A. 1983. A Broad Host Range Mobilization System for In Vivo Genetic Engineering: Transposon Mutagenesis in Gram Negative Bacteria. Nat Biotech 1:784-791.
2. Shin H, Mally M, Meyer S, Fiechter C, Paroz C, Zaehringer U, Cornelis GR. 2009. Resistance of Capnocytophaga canimorsus to killing by human complement and polymorphonuclear leukocytes. Infect Immun 77:2262-2271.

Table S3. Oligonucleotides used in this study

Ref.	Name	Sequence 5'-3'	Restriction	Gene
7958	wbuB6/9-KO1.1	GGCTGCAGAAGAATTTCCGAGGGCTGATG	*Pst*I	*wbuB*
7959	wbuB6/9-KO1.2	CTATGATGTTGCAAATACCGATGAGCACTTTT TATATTTCTTCGCT		*wbuB*
7962	wbuB6/9-KO3.1	AGCGAAGAAATATAAAAAGTGCTCATCGGTAT TTGCAACATCATAG		*ermF*
7975	wbuB6-KO3.2	AAACTCCTGTGATGATGGTTTCTACGAAGGAT GAAATTTTTCAGG		*ermF*
7974	wbuB6-KO2.1	CCTGAAAAATTTCATCCTTCGTAGAAACCATC ATCACAGGAGTTT		*wbuB*
7961	wbuB6/9-KO2.2	CCACTAGTAGGAATGCCTTTCTCTACTATC	*Spe*I	*wbuB*
7963	wbuB9-KO3.2	AAGCTCCTGTGATGATGATTTCTACGAAGGAT GAAATTTTTCAGG		*ermF*
7960	wbuB9KO2.1	CCTGAAAAATTTCATCCTTCGTAGAAATCATC ATCACAGGAGCTT		*wbuB*
8063	wbtA12-KO1.1	GGCTGCAGTCGCATATTGGGTACAACTATAG	*Pst*I	*wbtA*
8064	wbtA12-KO1.2	CTATGATGTTGCAAATACCGATGAGCTTTTGG TTAGTATAAAAATGG		*wbtA*
8067	wbtA12-KO3.1	CCATTTTTATACTAACCAAAAGCTCATCGGTA TTTGCAACATCATAG		*ermF*
8068	wbtA12-KO3.2	CCTTTCATAGTATTTAATATTCTACGAAGGAT GAAATTTTTCAGG		*ermF*
8065	wbtA12-KO-2.1	CCTGAAAAATTTCATCCTTCGTAGAATATTAA ATACTATGAAAGG		*wbtA*
8066	wbtA12-KO-2.2	CCACTAGTTCTCTTCGATGGAAAGTACG	*Spe*I	*wbtA*
8244	SeroA-fw	CATACCATGGGAAAAAAAGTACCAATAGTTTT TATATTTAACC	*NcoI*	*A4GalT GT*
8245	SeroA-rev	CCGCTCGAGTCATTTTTTTATCTTTTTTAATAT ATTCCAC	*XhoI*	*A4GalT GT*
8246	SeroB-fw	CATACCATGGGAATTAACAAAATTCTAATAG	*NcoI*	*GT1*
8247	SeroB-rev	CCGCTCGAGTTATTTTTTATTTTCATTAG	*XhoI*	*GT1*
8274	SeroC-fw	GGCGTATATCGTTGCTATTTTGTATG		*wzy*
8275	SeroC-rev	CTATTAATATTTTCATTGTACACCACTTC		*wzy*
8276	SeroD-fw	GATTTAAAAAATATAGTATTTTAGGAATTATCG		*WbbJ*
8277	SeroD-rev	CTATACTTGTTCCCACTTTTTAGTTTC		*WbbJ*
8278	SeroE-fw	GGAGGAGGAAAAGTATTATTAGATTATC		*GT1*
8279	SeroE-rev	CTATTCATAATTCTTAAAGATACTTATCAATTC		*GT1*
8296	SeroABC-fw	CTTGGTTAGGTAAAGTTGCCTTAC		*wfdR*
8297	SeroABC-rev	CAACATTTCTCCCATCTTATAATCCC		*wfdR*

Table S4. Accession numbers of genes used in this study

Serovar	Strain	Gene name	Locus	Accession
A	Cc5	*A4GalT GT*	*Ccan_23210*	NC_015846.1
A	Cc5	*wbuB*	*Ccan_23370*	NC_015846.1
A	Cc5	*wbtA*	*Ccan_23400*	NC_015846.1
A	Cc5	*wfdR*	*Ccan_23240*	NC_015846.1
A	Cc2	*A4GalT GT*	*CCAN2_1920004*	CDOJ01000104.1
A	Cc2	*GT1*	*CCAN2_1430008*	CDOJ01000050.1
A	Cc2	*wfdR*	*CCAN2_1430002*	CDOJ01000050.1
B	Cc6	*wbuB*	*Cc6_1430029*	LT838810
B	Cc6	*GT1*	*CC6_1430035*	LT838810
B	Cc6	*wfdR*	*CC6_1430040*	LT838810
B	Cc11	*GT1*	*CCAN11_10027*	CDOK01000001.1
B	Cc11	*wfdR*	*CCAN11_2010013*	CDOK01000115.1
C	Cc9	*wbuB*	*CCAN9_740038*	LT838811
C	Cc9	*wzy*	*CCAN9_740031*	LT838811
C	Cc9	*GT1*	*CCAN9_740032*	LT838811
C	Cc9	*wfdR*	*CCAN9_740027*	LT838811
D	Cc12	*wbtA*	*CCAN12_760057*	CDOE01000074.1
D	Cc12	*Wbbj*	*CCAN12_760043*	CDOE01000074.1
E	Cc4	*wbtA*	*CC4_530070*	LT838812
E	Cc4	*GT1*	*CC4_530066*	LT838812

Table S5: Capsular serovars distribution according to isolates geographical origin

Serovar	Geographical origin		Isolate ID
	Country	State and/or city	
A	Sweden	Malmö	Cc1
		Göteborg	Cc21
			Cc25
		Tröllhättan	Cc24
	Belgium	Brussels	Cc2
		Brugges	Cc3
		Libramont	Cc5
	USA	California	Cc10
	Netherlands	Roermond	Cc13
	Denmark	…	Cc22
	Switzerland	Bern	Cc15
B	Belgium	Leuven	Cc6
		Liège	Cc8
		Brussels	Cc16
			Cc17
			Cc18
	Switzerland	Zurich	Cc11
	France	Marseille	Cc23
C	USA	Virginia	Cc9
	Germany	Dresden	Cc14
	Belgium	Ottignies	Cc19
	UK	Colindale	Cc20
D	Belgium	Leuven	Cc7
	USA	California, San Antonio	Cc12
E	Switzerland	Geneva	Cc4
